# Custom-Made Total Temporomandibular Joint Replacement: A Long-Term Observational Case Series of Four Patients

**DOI:** 10.3390/jcm15197395

**Published:** 2026-09-23

**Authors:** Ramón Sieira-Gil, Ada Ferrer-Fuertes, Eloy García-Díez, Francisco Cuesta-González, Octavi Camps-Font, Eduard Valmaseda-Castellón, Carles Martí-Pagès

**Affiliations:** 1 Oral & Maxillofacial Surgery Department, Hospital Clínic de Barcelona, Universitat de Barcelona, 08036 Barcelona, Barcelona, Spain; ferrer@clinic.cat (A.F.-F.); eloy.garciadiez@gmail.com (E.G.-D.); fjcuesta@clinic.cat (F.C.-G.); martipages.c@gmail.com (C.M.-P.); 2 Facial Plastic & Orthognathic Surgery, SIES Maxillofacial Surgery, Clínica Planas, 08034 Barcelona, Barcelona, Spain; 3 Clínica Faces, 08021 Barcelona, Barcelona, Spain; 4 Department of Odontoestomatology, Faculty of Medicine and Health Sciences, Universitat de Barcelona, 08907 L’Hospitalet de Llobregat, Barcelona, Spain; eduardvalmaseda@ub.edu; 5 Bellvitge Biomedical Research Institute (IDIBELL), 08907 L’Hospitalet de Llobregat, Barcelona, Spain

**Keywords:** custom-made TMJ prostheses, temporomandibular disorders, temporomandibular joint reconstruction, TMJ replacement, orthognathic surgery

## Abstract

**Background/Objectives:** Advanced acquired or congenital temporomandibular joint (TMJ) pathology may severely impair oral function and quality of life, often requiring total joint replacement when conservative and reconstructive treatments fail. Custom-made TMJ prostheses (CMTMJP) have been developed to improve anatomical adaptation and functional outcomes. The aim of this study was to evaluate the medium- to long-term clinical outcomes of CMTMJP from Avinent^®^ in patients with advanced TMJ pathology requiring TMJ replacement. **Methods:** An observational follow-up study was conducted in patients treated with CMTMJP at the Department of Oral and Maxillofacial Surgery of Hospital Clínic and Barnaclínic (Barcelona, Spain). Clinical outcomes, including pain intensity assessed using a numerical rating scale, maximum interincisal opening (mm), and occlusal stability, were evaluated before and after surgery. Patients were followed for 50–70 months after implantation. **Results:** Functional improvement was maintained over time, with satisfactory pain control, stable occlusion, and adequate mandibular mobility. No CMTMJP failures, complications, or adverse events related to the prostheses were recorded. Surgeons reported favorable outcomes regarding the planning protocol, surgical placement, and postoperative management of the CMTMJP. **Conclusions:** CMTMJP replacement appears to be a predictable and reliable treatment option when the TMJ needs to be replaced for any cause, such as degenerative, acquired or congenital condition. The CMTMJP described in this series demonstrated good functional performance and long-term stability, with no major complications observed during 50–70 months of follow-up.

## 1. Introduction

Temporomandibular disorders (TMDs) comprise a heterogeneous group of conditions affecting the temporomandibular joint (TMJ), masticatory muscles, and associated craniofacial structures, either congenital or acquired. Epidemiological studies suggest that signs or symptoms of TMDs may affect up to 25% of the general population, although only a subset of individuals require treatment [1,2]. Clinical manifestations range from mild joint sounds and intermittent discomfort to severe pain, restricted mandibular mobility, occlusal instability, and substantial deterioration in oral function and quality of life. In advanced cases, Temporo mandibular pathology may impair mastication, speech, breathing, and swallowing, with significant physical, psychological, and social consequences [2,3,4].

The etiology for TMJ replacement is multifactorial and includes developmental abnormalities, traumatic injuries, inflammatory and degenerative joint diseases, autoimmune disorders, neoplastic conditions, previous TMJ surgery, and fibrous or bony ankylosis [5].

Historically, a variety of surgical approaches have been proposed for the management of end-stage TMJ disease and conditions where the TMJ is absent or needs to be reconstructed, including arthroplasty, autogenous graft reconstruction, costochondral grafting, and alloplastic joint replacement. However, long-term outcomes following autogenous reconstruction have often been limited by donor-site morbidity, unpredictable growth patterns, graft resorption, ankylosis, and relapse [5,6]. Consequently, total TMJ replacement has emerged as the preferred treatment modality for patients with severe joint destruction, ankylosis, advanced osteoarthritis, inflammatory arthropathies, or complex congenital and acquired deformities requiring TMJ reconstruction [3,7,8].

Over the last two decades, advances in computer-assisted planning, three-dimensional imaging, computer-aided design/computer-aided manufacturing (CAD/CAM), and additive manufacturing technologies have transformed the field of TMJ reconstruction. Custom-made TMJ prostheses (CMTMJP) systems allow precise adaptation to individual anatomy and facilitate simultaneous correction of associated skeletal deformities when required. Compared to stock prostheses, customized devices may improve prosthesis fit, reduce the need for intraoperative modifications, optimize load distribution, and enhance surgical predictability [5,9].

Current evidence consistently demonstrates that total TMJ replacement is associated with significant reduction in pain, improvement in mandibular function, increased maximal mouth opening, and enhanced quality of life [10,11,12,13]. Furthermore, several studies have reported substantial improvement in dietary function, social interaction, and patient satisfaction following reconstruction [14]. Nevertheless, outcomes may be influenced by factors such as the underlying pathology, the number of previous surgical interventions, and the complexity of the associated dentofacial deformity. In particular, with a history of multiple TMJ surgeries generally experience less substantial improvement in pain and mandibular function than those undergoing primary joint reconstruction [14].

The long-term success of CMTMJP also depends on the biomechanical and tribological properties of the implant materials. Contemporary CMTMJP typically combine titanium alloys, most commonly Ti-6Al-4V, with ultra-high-molecular-weight polyethylene (UHMWPE). These materials have demonstrated excellent biocompatibility, corrosion resistance, fatigue strength, and wear performance, making them suitable for long-term articulation under repetitive functional loading [5,15]. Continuous improvement in material processing and surface engineering have further contributed to enhancing prosthetic durability and reducing wear-related complications.

Despite the growing adoption of CMTMJP, clinical evidence remains relatively limited, particularly regarding medium- and long-term outcomes of newer customized systems. Therefore, the aim of the present study was to evaluate the clinical performance of a CMTMJP from Avinent^®^ (Avinent Implant System, Barcelona, Spain) by describing pain outcomes, mandibular function, occlusal stability, patient-reported outcomes, and complications in a consecutive series of patients followed for up to six years after surgery.

## 2. Materials and Methods

### 2.1. Study Design and Ethical Considerations

The present retrospective observational case series was conducted between 2017 and 2023 at the Department of Oral and Maxillofacial Surgery, Hospital Clínic–Barnaclínic (Barcelona, Spain). Four patients requiring total TMJ replacement were treated between September 2017 and May 2019, resulting in the implantation of five CMTMJP of the Avinent System^®^.

During this period more than 60 cases have been treated for TMJ replacement including total and partial TMJ replacement due to oncological, acquired or congenital conditions. We report the cases treated under this new system as a small case series observational study.

The study protocol was approved by the local Ethics Committee (Protocol No. HCB/2021/0570) and was conducted in accordance with the principles of the Declaration of Helsinki for research involving human subjects [16]. All participants received detailed information regarding the study objectives, procedures, and potential risks and provided written informed consent before enrollment. The case series was reported in accordance with the CARE guidelines (Supplementary File S1).

### 2.2. Intervention and Postoperative Care

All procedures were performed by senior surgeons from the Department of Oral and Maxillofacial Surgery at Hospital Clínic–Barnaclínic (Barcelona, Spain).

Surgical protocol was adapted to each diagnosis. When resection of the impaired TMJ was needed, custom made surgical guides of acrylic were used for a guided resection of the fossa and the condyle together with the coronoid process. When the TMJ was not present, a surgical guide was used for the surgical site of the fossa preparation and another guide for the mandibular ramus osteotomy if needed.

A classical extended Al-Kayat Bramley and Risdon submandibular approach was done for the site of CMTMJP. An intra-oral approach was performed in the cases with concomitant orthognathic surgery. A mandibular first protocol was done in all cases when concomitant orthognathic surgery was performed IMF screws were used together with a surgical occlusal splint to set the occlusion either at the intermediate or final occlusal position in all cases.

CMTMJP (Avinent^®^ Patient-Specific TMJ; Avinent Implant System, Barcelona, Spain) were used in all cases. Each prosthesis consisted of a two-component metal-on-polyethylene articulation comprising a patient-specific fossa component manufactured from UHMWPE and a mandibular ramus-condyle component fabricated from Ti-6Al-4V ELI titanium alloy (Figure 1, Figure 2 and Figure 3). A carbon-based surface treatment was applied to the metallic titanium component at the titanium–polymer interface to reduce friction. The UHMWPE component was compression-molded, highly cross-linked (approximately 75 kGy), and stabilized through post-irradiation thermal annealing.

Perioperative antibiotic prophylaxis, analgesics, and anti-inflammatory medications were administered according to institutional protocols. Surgical drains, when used, were removed within 24–48 h, and skin sutures were removed after 7–10 days.

Postoperative rehabilitation included supervised physiotherapy initiated within 24–72 h after surgery to promote early mandibular mobilization. Patients subsequently followed a structured home exercise program consisting of active and passive mandibular opening, closing, and lateral excursion exercises performed several times daily for a minimum of six weeks. A soft diet was recommended for 4–6 weeks, with gradual progression according to individual tolerance. Occlusal stability was assessed at each follow-up visit, and adjustments were performed when required.

First radiological follow up was performed immediately after the surgery during hospital stay. Then a CT scan was performed in the first month and in the third month during the follow up.

### 2.3. Data Collection and Outcome Measures

Clinical data were prospectively collected using standardized electronic case report forms as part of a predefined departmental follow-up protocol. Assessments were performed at the following time points: preoperative baseline (T_0_), intraoperative evaluation (T_1_), 2 months postoperatively (T_2_), 6 months (T_3_), 1 year (T_4_), 2 years (T_5_), and the most recent follow-up visit in 2023 (T_6_).

The outcomes included pain, maximum interincisal opening (MIO), postoperative occlusal stability, prosthesis-related complications, surgeon-reported intraoperative performance were reviewed in a retrospective manner. Pain was measured using an 11-point Numeric Rating Scale (NRS), ranging from 0 (no pain) to 10 (worst imaginable pain) [17]. Pain assessments were obtained at each follow-up visit to evaluate the evolution of symptoms over time. MIO was measured in millimeters as the distance between the incisal edges of the maxillary and mandibular central incisors during maximum unassisted mouth opening using a rigid ruler. Occlusal stability was assessed clinically at each follow-up visit, and any postoperative occlusal adjustments or corrective procedures were recorded.

Intraoperative variables included prosthesis adaptation to the recipient anatomy, the need for intraoperative modifications, fixation performance, and overall ease of use as reported by the operating surgeons. Device usability was evaluated with regard to implant handling, fitting accuracy, fixation, labeling, and clarity of the instructions for use.

Safety outcomes included the occurrence of intraoperative and postoperative complications, implant-related adverse events, prosthesis failure, component fracture, loosening, infection, and the need for revision or replacement surgery throughout the follow-up period.

All assessments were performed according to a standardized protocol by three members of the surgical team. Whenever feasible, evaluators were blinded to measurements obtained at previous time points to minimize assessment bias.

### 2.4. Statistical Analysis

Given the exploratory nature of this case series, the limited sample size, and the absence of a comparison group, all analyses were descriptive. The primary aim was to characterize the clinical performance and safety profile of the prostheses over time rather than to establish causal associations or test formal hypotheses. Accordingly, no inferential statistical analyses, multivariable modeling, adjustment for potential confounding variables, or formal hypothesis testing were performed.

Continuous variables were summarized using medians, interquartile ranges (IQRs), ranges, and individual patient values where appropriate. Categorical variables were reported as absolute frequencies and percentages.

Longitudinal outcomes, including pain intensity and MIO, were evaluated descriptively across the predefined follow-up time points (T_0_–T_6_). Individual patient trajectories were examined to assess temporal trends and the consistency of clinical outcomes throughout follow-up. Prosthesis-related complications, adverse events, revision procedures, and intraoperative usability outcomes were also analyzed descriptively and reported on a per-patient and per-prosthesis basis.

Missing data were not imputed, and analyses were based on all available observations at each follow-up interval.

### 2.5. Use of Generative Artificial Intelligence

During the preparation of this manuscript, the authors used ChatGPT (GPT-5.6 Sol, OpenAI, San Francisco, CA, USA) for language editing and improvement in scientific English. The tool was not used for study design, data collection, statistical analysis, interpretation of the results, or generation of scientific conclusions. All AI-generated output was reviewed and edited by the authors, who take full responsibility for the content of the manuscript.

## 3. Results

Four patients underwent CMTMJP of the Avinent System^®^ between September 2017 and May 2019. Three patients underwent unilateral replacement and one underwent bilateral replacement, resulting in the implantation of five CMTMJP (Avinent^®^ Patient-Specific TMJ; Avinent Implant System, Barcelona, Spain). Follow-up ranged from 50 to 70 months. Surgical indications are summarized in Table 1.

At the first postoperative visit, three minor complications unrelated to the CMTMJP were recorded, all occurring contralateral to the implant side. All events resolved following antibiotic therapy (Table 2). No prosthesis-related adverse events, implant removals, or revision surgeries were required during follow-up. No intraoperative or postoperative technical complications involving the prosthetic components or fixation screws were observed, and no component fractures occurred. In all cases, the prostheses demonstrated an adequate fit to the recipient anatomy without the need for intraoperative modification. Intraoperative usability, including device handling, labeling, and instructions for use, was rated as satisfactory in all procedures. Figure 4 shows the preoperative presentation of a representative case, and Figure 5 the postoperative outcome at final follow-up. Figure 6 shows the postoperative CT scan performed 1 month after surgery.

Pain outcomes are presented in Table 3. NRS data was available for three patients (four joints), as complete recordings were unavailable for one patient (one joint). Preoperative pain levels were low (Median: 1; Range: 0 to 4). At 2 months postoperatively (T_2_), transient pain was reported in three joints (NRS scores of 1–2, 5, and 7), whereas one joint remained pain-free. From 6 months onward (T_3_–T_6_), all evaluated joints reported an NRS score of 0 at every follow-up assessment.

Median MIO was 32 mm (IQR: 5 mm) at both the 2-year (T_5_) and final follow-up (T_6_) evaluations (Table 4). At the last follow-up visit, three patients exhibited a stable Class I occlusion. One patient (Case 3) maintained residual occlusal asymmetry because concomitant maxillary orthognathic surgery could not be performed at the time of TMJ replacement.

No clinical or technical complications attributable to prosthesis malfunction, fixation failure, wear, loosening, infection, or biologic failure were observed during the follow-up period. Consequently, no device removal or replacement procedures were required.

## 4. Discussion

The present case series evaluated the medium-term clinical performance of CMTMJP Avinent System^®^ in patients requiring TMJ replacement. The principal findings were the absence of device-related complications, prosthesis failures, or revision surgeries during 50–70 months of follow-up, together with sustained pain relief, stable mandibular function, and satisfactory occlusal outcomes. These findings suggest that CMTMJP Avinent System^®^ may provide predictable functional rehabilitation and durable clinical stability in carefully selected patients.

The use of CMTMJP has increased over the last decade owing to advances in virtual surgical planning, computer-aided design, and additive manufacturing technologies. Compared with stock prostheses, customized systems are designed to reproduce patient anatomy more accurately, potentially improving prosthesis adaptation, minimizing intraoperative modifications, and facilitating surgical workflow. In the present series, all CMTMJP demonstrated an adequate fit without requiring intraoperative adjustment, supporting previous reports that have highlighted the accuracy and predictability of CMTMJP [6,9,18,19].

Functional rehabilitation was favorable. At the latest follow-up, MIO reached a median value of 32 mm, which compares favorably with values reported in previous studies of both customized and stock TMJ prostheses. Although greater mouth-opening values have been reported in some cohorts, restoration of painless mandibular function and maintenance of an adequate oral opening for daily activities may be more clinically meaningful than achieving maximal opening alone. In the present cohort, MIO did not increase substantially in absolute terms; rather, functional mouth opening was maintained throughout follow-up, as shown in Table 4. Furthermore, occlusal stability was preserved in three of the four patients. The residual asymmetry observed in one patient was attributable to incomplete correction of the associated dentofacial deformity rather than to prosthetic failure, underscoring the importance of comprehensive treatment planning in patients requiring concomitant orthognathic procedures.

Pain reduction represents an important treatment goal of total TMJ replacement in patients presenting with preoperative pain. Although transient postoperative pain was observed during the early healing period, all evaluated joints were pain-free from 6 months onward and remained asymptomatic throughout the subsequent follow-up period. This pattern is consistent with previous studies reporting substantial reductions in pain following total TMJ replacement, with improvements typically becoming more evident after completion of postoperative rehabilitation and functional adaptation to the CMTMJP [12]. Although the low preoperative pain scores observed in our cohort limited the magnitude of measurable improvement and may have introduced a floor effect, the complete absence of pain at long-term follow-up remains a clinically relevant finding.

The safety profile of the prosthetic system was favorable with respect to device-related complications. No implant-related infections, fixation failures, component fractures, loosening events, or prosthesis removals occurred during follow-up. However, postoperative complications were not uncommon, as two of the four patients developed infections at the contralateral osteotomy site following concomitant orthognathic surgery. These infections were unrelated to the CMTMJP and resolved with conservative treatment without compromising prosthesis function or requiring revision surgery. The absence of prosthesis-related adverse events is consistent with previous reports demonstrating low rates of mechanical complications and revision surgery following the use of modern CMTMJP as the one evaluated in this study [9,10].

The present study has several limitations. First, the small sample size, inherent to both the rarity of the indication and the single-center design, limits the generalizability of the findings and precludes inferential statistical analyses. Accordingly, the results should be interpreted as descriptive and hypothesis-generating. Second, the absence of a control group precludes direct comparison with alternative treatment modalities or stock prosthetic systems. Third, some patient-reported outcome measures were unavailable at the early follow-up visits because these variables were not included in the standardized data collection protocol at the beginning of the study period. Although clinical data were prospectively collected using standardized electronic case report forms, this retrospective analysis was necessarily restricted to the variables available in the original dataset, limiting the assessment of perioperative quality-of-life changes. Finally, outcome assessments were performed by members of the treating team, introducing the potential for observer bias.

Despite these limitations, the study contributes medium-term clinical data on a relatively novel CMTMJP system and demonstrates favorable outcomes in terms of prosthesis survival, pain control, functional rehabilitation, and safety. Future prospective multicenter studies with larger sample sizes, standardized patient-reported outcome measures, and longer follow-up periods are needed to confirm these findings and to better characterize long-term prosthesis performance and survivorship.

## 5. Conclusions

Custom-made temporomandibular joint prostheses were associated with maintenance of functional mouth opening and favorable pain outcomes over follow-up periods of up to six years. Given the small sample size and observational design of this case series, these findings should be interpreted with caution and require confirmation in larger prospective studies with standardized long-term follow-up.

## Figures and Tables

**Figure 1:**
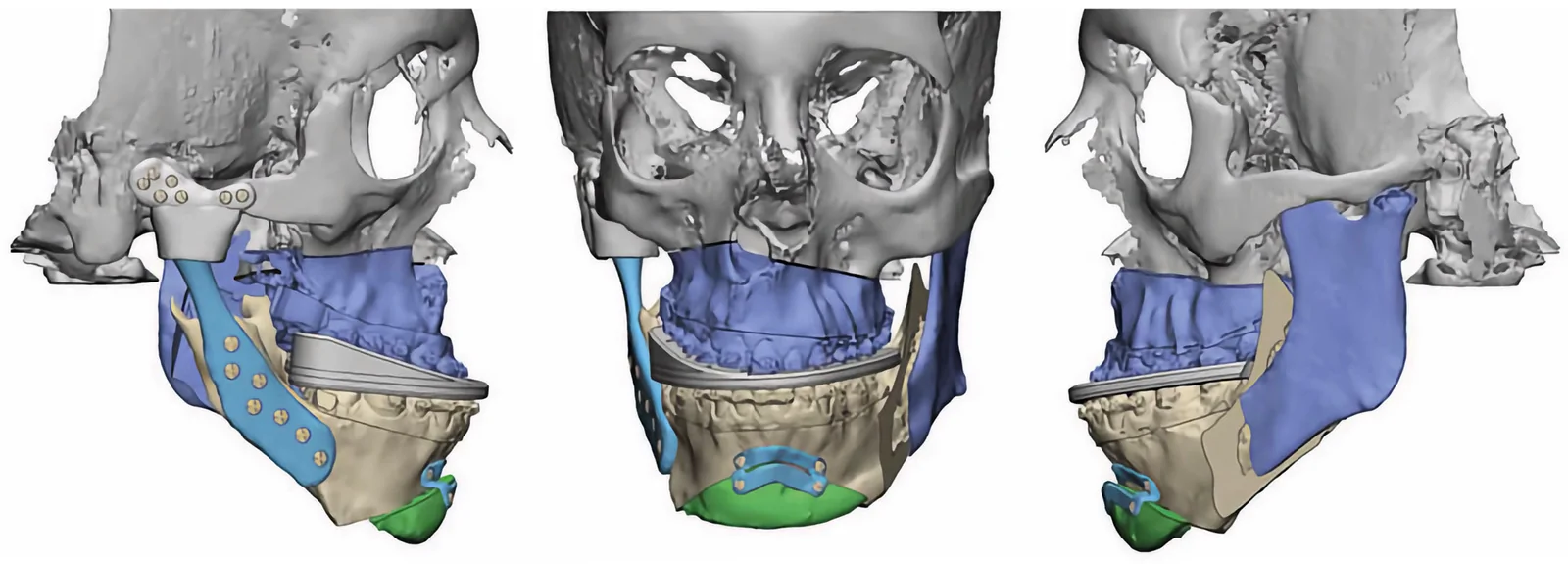
Intermediate surgical planning with the mandible in the final position with the Custom-Made Temporo Mandibular Joint Prosthesis Avinent System^®^, the guided genioplasty performed with a custom made titanium plate, and the maxilla in the initial position.

**Figure 2:**
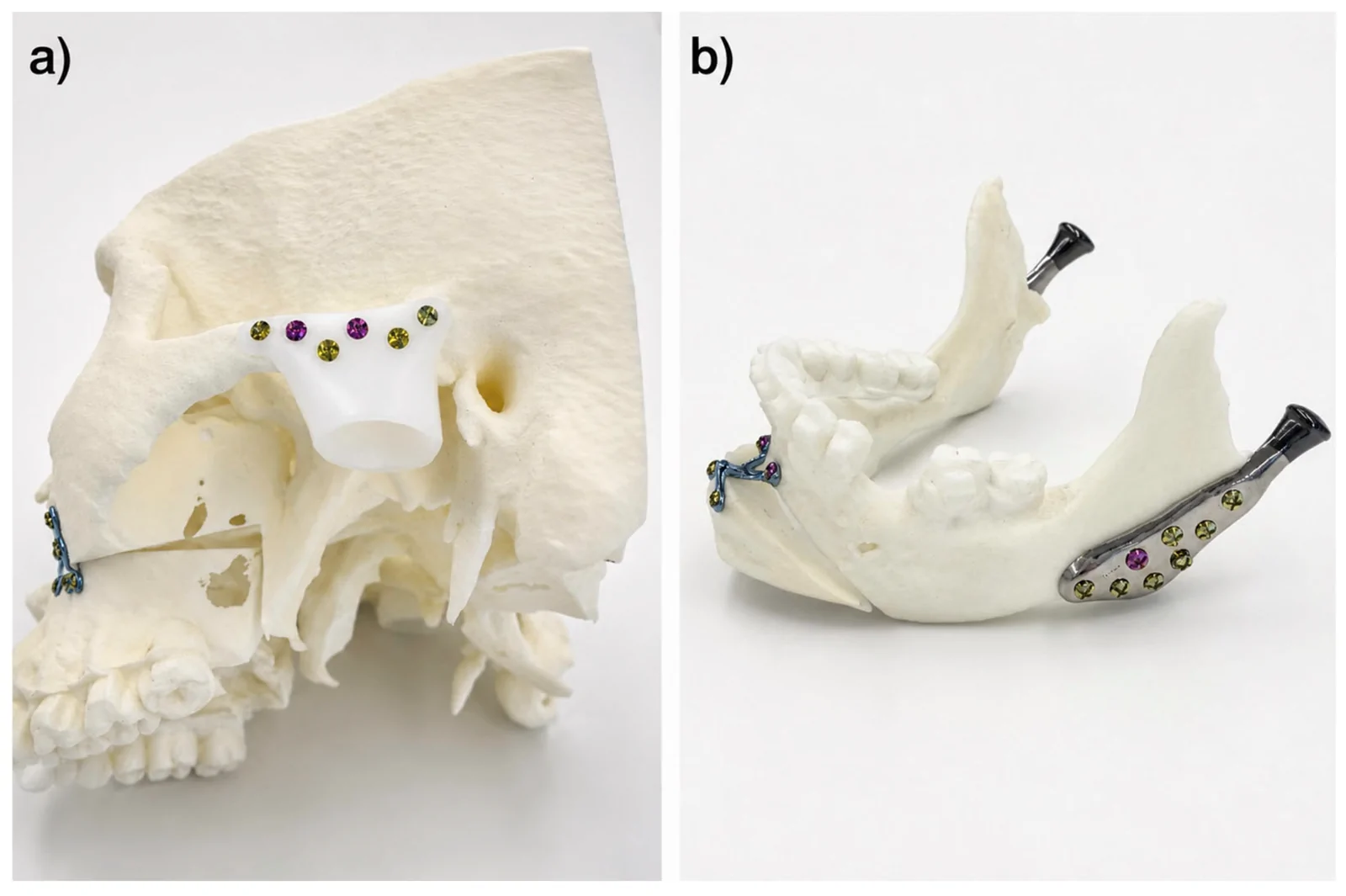
Custom-Made Temporo Mandibular Joint Prosthesis Avinent System^®^: (**a**) detail of the fossa component manufactured from ultra-high-molecular-weight polyethylene; (**b**) detail of the mandibular ramus–condyle component manufactured from Ti-6Al-4V extra-low-interstitial titanium alloy.

**Figure 3:**
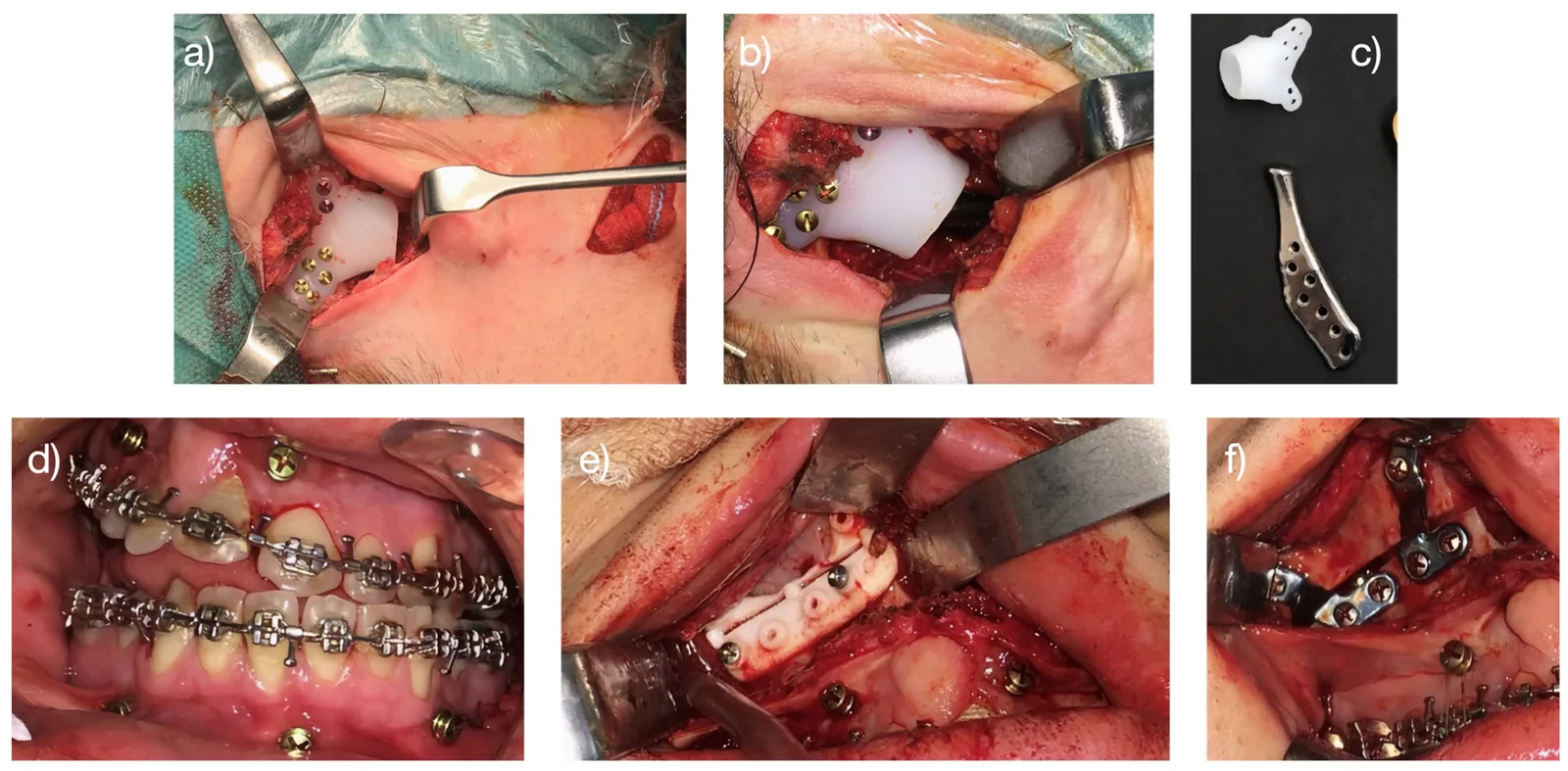
Intraoperative photographs illustrating temporomandibular joint prosthesis placement. (**a**) Detail of the UHMWPE Fossa Component of the CMTMJP; (**b**) Detail of the position of the Condyle component of the CMTMJP in the fossa component; (**c**) Detail of the UHMWPE Fossa component and the mandibular component made of titanium alloy; (**d**) Intermediate position after TMJ prosthesis placement and contralateral sagital split osteotomy. The position matches with the planned intermediate position; (**e**) Intraoperative detail of surgical guide for the Le Fort osteotomy; (**f**) Intraoperative detail of the custom made osteosynthesis for the Le Fort rigid fixation.

**Figure 4:**
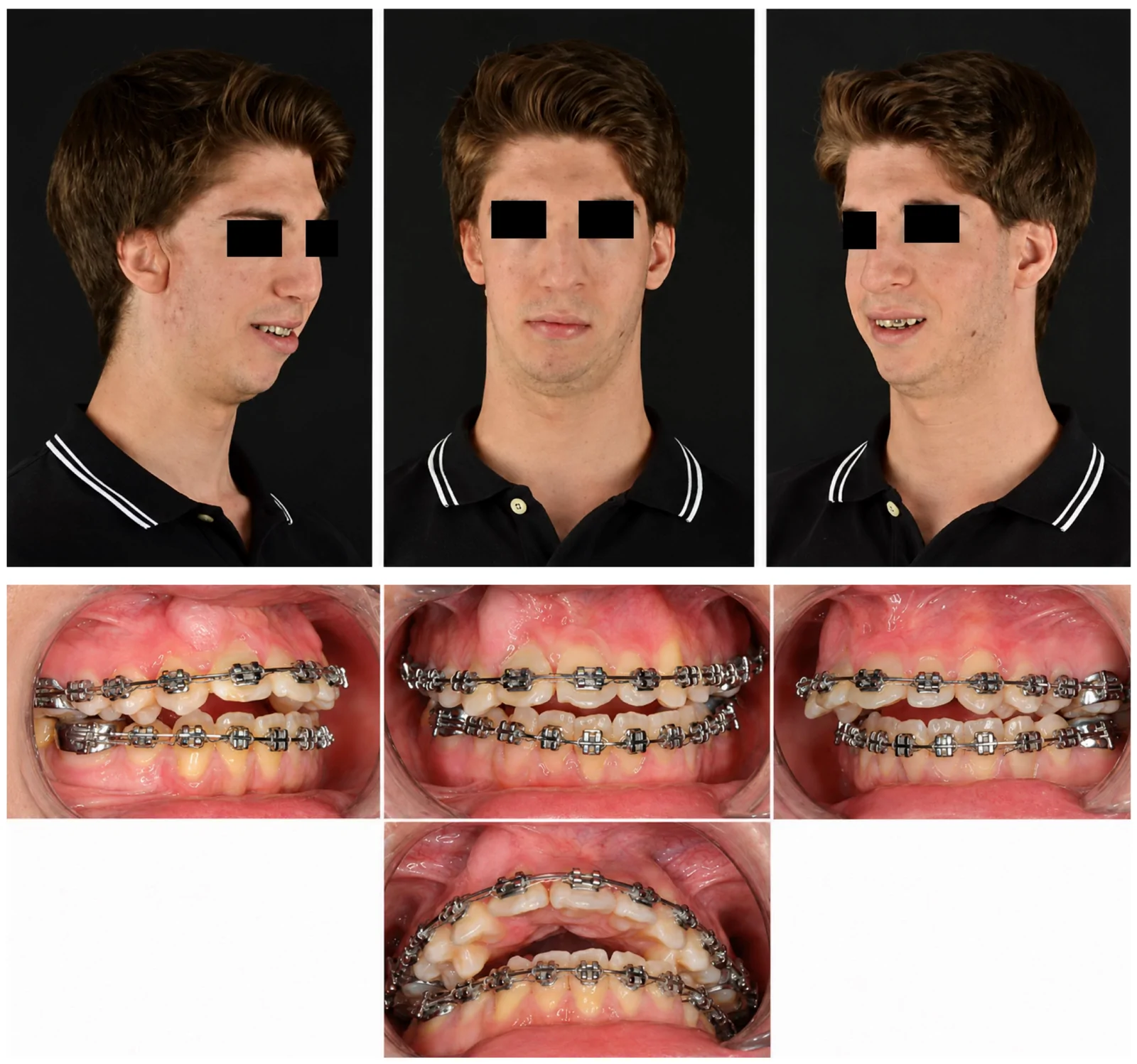
Preoperative facial pictures and Intraora preoperative orthodontic preparation for simultaneous orthognathic surgery and Custom-Made Temporo Mandibular Joint Prosthesis.

**Figure 5:**
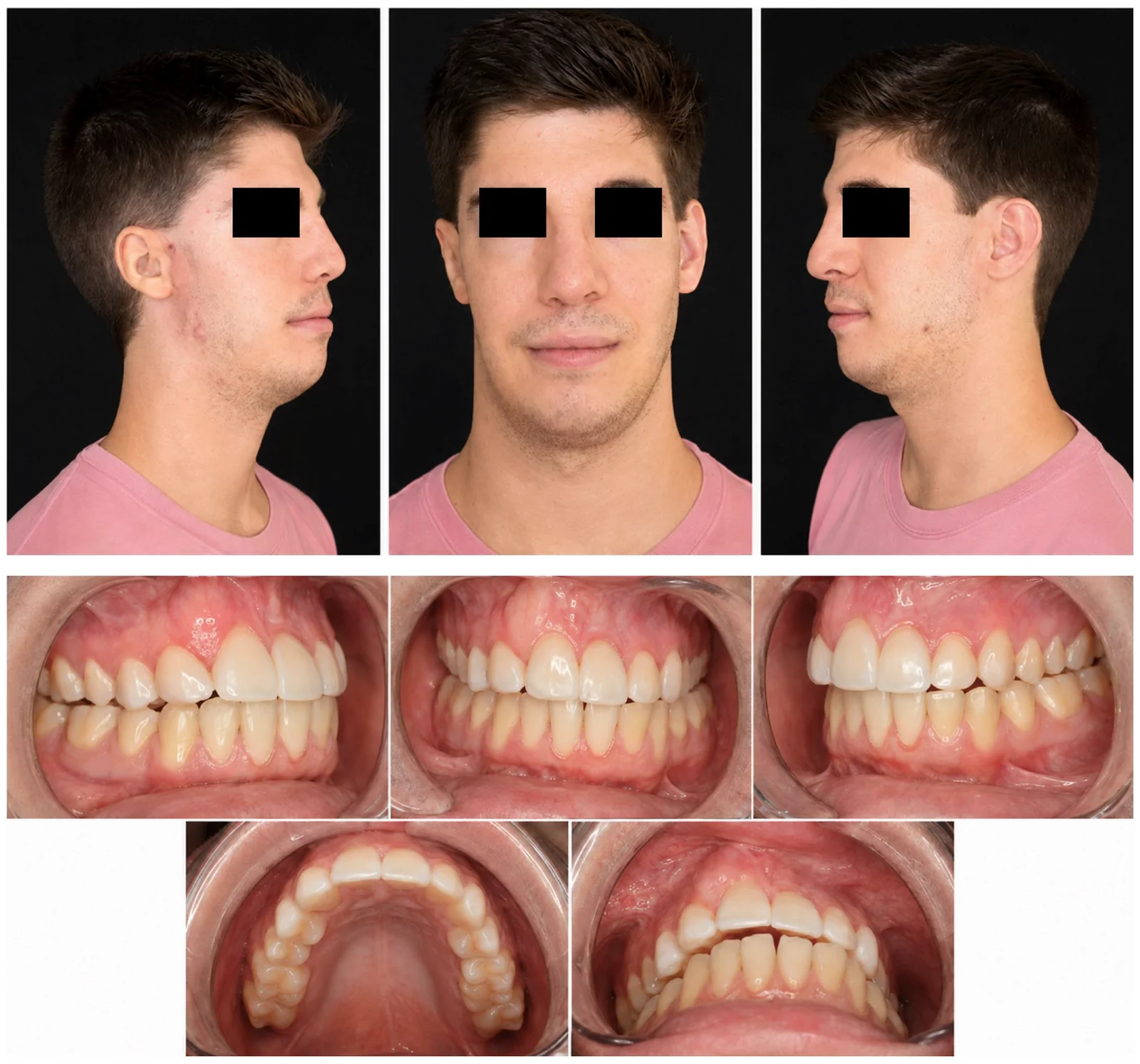
Postoperative facial appearance 9 months after concomitant orthognathic surgery and custom-made temporomandibular joint prosthesis placement, and 3 months after rhinoplasty with rib grafting. Postoperative intraoral and occlusal appearance, including cosmetic dental treatment, 6 months after concomitant orthognathic surgery and CMTMJP placement.

**Figure 6:**
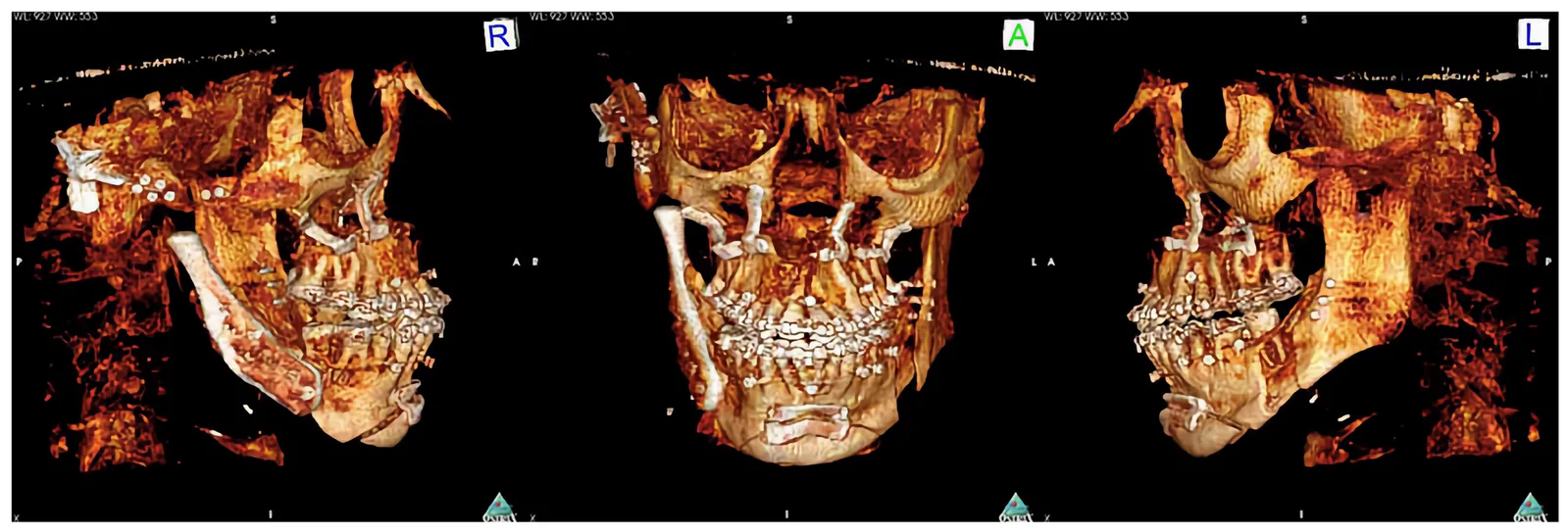
Postoperative computed tomography showing the radiographic outcome after concomitant orthognathic surgery and custom-made temporomandibular joint prosthesis placement. R: right side. A: anterior view. L: left side.

**Table 1:** Demographic characteristics and implant indications of the patients treated with Custom-Made Temporo Mandibular Joint Prostheses.

Case	Age	Sex	Side	Diagnosis	Previous Pain	Functional Limitation	Previous TMJ Surgery
1	48	Female	Bilateral	Degenerative bone disease	1–2 Mild	Yes	No
2	40	Female	Unilateral	Obstructive sleep apnea syndrome Condylar resorption	3–6 Moderate	Yes	No
3	36	Male	Unilateral	Congenital malformation Right hemifacial microsomia	1–2 Mild	Yes	No
4	24	Male	Unilateral	Congenital malformation Goldenhar syndrome	0 No pain	Yes	No

**Table 2:** Summary of the recorded postsurgical clinical complications.

Case	Adverse Event	Product Defect	Infection	Other
1	No	No	No	External otitis
2	No	No	No	Contralateral osteotomy infection
3	No	No	No	Contralateral osteotomy infection
4	No	No	No	No

**Table 3:** Numerical pain scores over patient follow-up.

Case	Pain Score T_0_	Pain Score T_2_	Pain Score T_3_	Pain Score T_4_	Pain Score T_5_	Pain Score T_6_
1	1	7	0	0	0	0
2	4	1–2	0	0	0	0
3	1	5	0	0	0	0
4	0	0	0	0	0	0

T_0_ Pre-surgery control; T_1_ Surgical phase; T_2_ Follow-up at 2 months post-surgery; T_3_ Follow-up at 6 months post-surgery; T_4_ Follow-up at 1 year post-surgery; T_5_ Follow-up at 2 years post-surgery; and T_6_ current control.

**Table 4:** Maximum interincisal opening (MIO) at the different timepoints over follow-up.

Case	MIO T_0_	MIO T_2_	MIO T_3_	MIO T_4_	MIO T_5_	MIO T_6_
1	32	17	35	33	32	32
2	35	35	32	32	32	32
3	50	35	52	52	37	37
4	40	40	40	40	40	40

T_0_ Pre-surgery control; T_1_ Surgical phase; T_2_ Follow-up at 2 months post-surgery; T_3_ Follow-up at 6 months post-surgery; T_4_ Follow-up at 1 year post-surgery; T_5_ Follow-up at 2 years post-surgery; and T_6_ current control.

## Data Availability

The data presented in this study are available from the corresponding authors upon reasonable request.

## Supplementary Materials

The following supporting information can be downloaded at: https://www.mdpi.com/article/10.3390/jcm15197395/s1, File S1. CARE Checklist of information to include when writing a case report.

## Abbreviations

The following abbreviations are used in this manuscript:

CMTMJP	Custom-Made Temporo Mandibular Joint Prosthesis
CAD/CAM	Computer-Aided Design/Computer-Aided Manufacturing
MIO	Maximum Interincisal Opening
NRS	Numeric Rating Scale
TMJ	Temporomandibular Joint
UHMWPE	Ultra-High-Molecular-Weight Polyethylene
Ti-6Al-4V ELI	Extra-Low Interstitial Titanium Alloy (Titanium–6% Aluminum–4% Vanadium)
