# Supplementary material for: Custom-Made Total Temporomandibular Joint Replacement: A Long-Term Observational Case Series of Four Patients
Source: J Clin Med. 2026 Sep 23;15(19):7395. doi: 10.3390/jcm15197395 (PMC13665894; doi:10.3390/jcm15197395)
Supplement: Supplementary file 1 [file jcm-15-07395-s001.zip › jcm-4463695-supplementary.pdf]

CARE case report guidelines

CARE Checklist of information to include when writing a case report

(cc) BY-NC-ND

Topic	Item	Checklist item description	Reported on Line
Title	1	The diagnosis or intervention of primary focus followed by the words "case report"	2-4 (case series)
Key Words	2	2 to 5 key words that identify diagnoses or interventions in this case report, including "case report"	43-44 (no case report)
Abstract (no references)	3a	Introduction: What is unique about this case and what does it add to the scientific literature?	NR
	3b	Main symptoms and/or important clinical findings	33-37
	3c	The main diagnoses, therapeutic interventions, and outcomes	27-37
	3d	Conclusion—What is the main "take-away" lesson(s) from this case?	38-42
Introduction	4	One or two paragraphs summarizing why this case is unique (**may include references**)	95-104
Patient Information	5a	De-identified patient specific information.	Table 1: 255-264
	5b	Primary concerns and symptoms of the patient.	Table 1: 255-264
	5c	Medical, family, and psycho-social history including relevant genetic information	NR
	5d	Relevant past interventions with outcomes	255-264 (partial)
Clinical Findings	6	Describe significant physical examination (PE) and important clinical findings.	255-264 (partial)
Timeline	7	Historical and current information from this episode of care organized as a timeline	179-184 (follow-up)
Diagnostic Assessment	8a	Diagnostic testing (such as PE, laboratory testing, imaging, surveys).	NR
	8b	Diagnostic challenges (such as access to testing, financial, or cultural)	NR
	8c	Diagnosis (including other diagnoses considered)	Table 1: 255-264
	8d	Prognosis (such as staging in oncology) where applicable	N/A
Therapeutic Intervention	9a	Types of therapeutic intervention (such as pharmacologic, surgical, preventive, self-care)	121-143; 163-174
	9b	Administration of therapeutic intervention (such as dosage, strength, duration)	121-143; 163-174
	9c	Changes in therapeutic intervention (with rationale)	173-174 (partial)
Follow-up and Outcomes	10a	Clinician and patient-assessed outcomes (if available)	242-299
	10b	Important follow-up diagnostic and other test results	175-177; 284-286
	10c	Intervention adherence and tolerability (How was this assessed?)	NR
	10d	Adverse and unanticipated events	242-259; 296-299
Discussion	11a	A scientific discussion of the strengths AND limitations associated with this case report	348-365
	11b	Discussion of the relevant medical literature **with references**.	308-347
	11c	The scientific rationale for any conclusions (including assessment of possible causes)	301-347
	11d	The primary "take-away" lessons of this case report (without references) in a one paragraph conclusion	369-374
Patient Perspective	12	The patient should share their perspective in one to two paragraphs on the treatment(s) they received	NR
Informed Consent	13	Did the patient give informed consent? Please provide if requested	Yes ☒ No ☐ Consent: 385-387
